# Mechanisms underlying the effects of nitrogen and phosphorus on the growth and berberine biosynthesis of *Phellodendron chinense* Schneid

**DOI:** 10.3389/fpls.2025.1704035

**Published:** 2025-12-10

**Authors:** Le Yang, Yuanzheng Gu, Hanjie He, Guangjun Wang, Rashad Qadri, Wende Yan, Gongxiu He, Rui Zhou

**Affiliations:** 1 State Key Laboratory of Utilization of Woody Oil Resource, Central South University of Forestry and Technology, Changsha, China; 2 Hunan Provincial Key Laboratory of Forestry Biotechnology and International Cooperation Base of Science and Technology Innovation on Forest Resource Biotechnology, Central South University of Forestry and Technology, Changsha, China; 3 National Engineering Laboratory for Applied Technology of Forestry and Ecology in South China, Central South University of Forestry and Technology, Changsha, China; 4 Institute of Horticultural Sciences, University of Agriculture, Faisalabad, Pakistan; 5 Hunan Shuangmu Pharmacy Limited Company, Longshan County, Xiangxi Tujia and Miao Autonomous Prefecture, Hunan, China

**Keywords:** *Phellodendron chinense* Schneid, berberine, nitrogen and phosphorus, soil microorganism, soil metabolism, gene expression

## Abstract

**Introduction:**

*Phellodendron chinense* Schneid (*P. chinense* Schneid) is a woody medicinal herb valued for its production of the bioactive compound berberine. Both its growth and berberine biosynthesis are regulated by nitrogen (N) and phosphorus (P) availabilities. However, the role of N and P in regulating rhizosphere soil, microenvironments, and microbial interactions remains unclear.

**Methods:**

To investigate regulatory effects of N and P on *P. chinense* Schneid seedlings, a seedbed experiment was conducted using five nutrient treatments: a control (CK), N alone (N10), and N combined with three levels of P (N10P5, N10P10, and N10P15). This study assessed changes such as pH, SOM, TN, and TP in soil properties, microbial communities, soil enzyme activities, plant biomass, berberine content, gene expression profiles, and metabolite content.

**Results:**

Experimental results demonstrated that, compared to the N10 treatment, combined N and P addition (N10P5, N10P10, and N10P15) significantly reduced the soil pH, soil organic matter (SOM), total nitrogen (TN), total phosphorus (TP), available phosphorus (AP) and potassium (AK), and bacterial α-diversity, suppressed the enzymatic activities in carbon and nitrogen metabolisms, whereas significantly increased the relative abundance of microbial taxa in the soil of *P. chinense* Schneid seedlings. Additionally, the combined treatments, especially N10P10 increased plant height by 83.80%, biomass by 82.07%, and berberine content by 13.57%. Correspondingly, expression analysis revealed that 5 metabolites including estrone and N-acetyl-L-leucine, etc., as well as the expression of 8 genes (e.g., *gdhA, smtA*, and *sga*) were upregulated in soil of *P. chinense* Schneid seedlings.

**Discussion:**

This study revealed a novel rhizosphere-mediated mechanism for N/P interaction in medicinal plants. We have found that N10P10 level significantly changes the soil microenvironment. These changes were related to the alterations in key functional gene expression and metabolic profiles, which promoted biomass growth and enhanced berberine synthesis in *P. chinense* Schneid seedlings. Therefore, it provides a concrete agronomic strategy: applying a balanced N:P ratio of 10:10 can be recommended to simultaneously maximize both the biomass and medicinal quality of *P. chinense* Schneid in cultivation.

## 1 Introduction

Nitrogen (N) and phosphorus (P) are important components of amino acids, plant hormones and alkaloids, and play a key role in plant growth and secondary metabolism (Baker et al., 2024; Fan et al., 2020; Liu F. et al., 2025; Liu YJ. et al., 2023; Xudong et al., 2022). Adding N and P fertilizers to the soil promotes the accumulation and metabolic capacity of N and P elements within cells, thereby promoting plant growth and the synthesis of secondary metabolites. Addition of appropriate concentrations of N and P increases the content of available N and P in the soil, which is directly absorbed, transported, and utilized by the root system, and thereby promoting plant growth (Zhang Y. et al., 2024). The addition of N and P promotes the synthesis of intracellular L-glutamate-tRNA, which in turn enhances the synthesis and accumulation of chlorophyll, the activity and efficiency of photosynthetic enzymes, the synthesis of ATP, the distribution of carbohydrate reserves and sources, and ultimately promotes plant growth (Yue et al., 2023). Simultaneously, N and P supplementation enhances secondary metabolism by promoting the synthesis of precursors (e.g., phenylalanine, tyrosine) and upregulating key pathway genes. These actions thereby increase the biosynthesis of bioactive alkaloids, flavonoids, terpenoids, and saponins. (Liu S. et al., 2025).

The N and P required for plant growth mainly come from the soil. In agricultural soils, a significant proportion of N and P exists in metal-bound forms. For instance, over 60% of P in acidic soils is stabilized by adsorption to Fe/Al oxides or precipitation as Fe/Al/Ca phosphates, while N is immobilized in organo-mineral complexes or adsorbed to Fe/Al oxides (Fang et al., 2024; Shi et al., 2025; Zhao et al., 2025). The application of N and P increases the content of available N and P in the soil in a short period of time, leading to soil acidification, promoting the degradation of soil organic matter, and inhibiting the activity of enzymes related to carbon, N and P metabolism. This shift in the soil physicochemical environment creates a new selective pressure that directly shapes the microbial habitat. However, as these nutrients are absorbed and utilized by plants, the content of total N, total P, organic matter, and other substances in the soil gradually decreases (Hu et al., 2024; Liu SX. et al., 2023; Ma et al., 2023). The addition of N and P altered the survival strategies of bacteria, inhibiting the proliferation of acid-sensitive groups and pathogens, reducing bacterial diversity, and regulating bacterial community composition and metabolic functions (Hu et al., 2024). Although combined N and P addition has no significant effect on soil fungal diversity, it regulates fungal community structure and metabolic function (Widdig et al., 2020). Moreover, N and P supplementation modified the relative abundance of key soil microorganisms, influencing the expression of phosphorus-responsive genes like *phoB* and *phoU*, as well as phosphorus solubilization-related genes such as *phoC*, *phoD*, and *phoA*. This regulation promoted the accumulation of N containing metabolites, including amino acids, nucleotides, quaternary ammonium compounds, and serotonin, while decreasing the levels of metabolites like organic acids, pentoses, and their corresponding alcohol derivatives. Serotonin has shown a significant positive correlation with the *Actinobacteria* phylum, indicating that combined N and P addition regulate the synthesis and accumulation of soil metabolites by modulating the abundance of key microorganisms and the expression of their genes (Baker et al., 2024; Liu S. et al., 2025). Crucially, this microbiome-driven regulating of the rhizosphere metabolic environment is hypothesized to provide a critical link to plant secondary metabolism.

*P. chinense* Schneid is a perennial deciduous tree belonging to the genus *Phellodendron* in the *Rutaceae* family, widely distributed in subtropical regions of China. As an important traditional medicinal plant, it contains active components such as berberine and flavonoids, and is widely used in the clinical treatment of diseases such as cancer, hepatitis, pneumonia, and diabetes (Gu et al., 2023; He et al., 2020; Yan et al., 2016; Yang et al., 2023; Zhang et al., 2023). Previous studies have shown that N10 treatment modulates rhizosphere microbial composition and nutrient cycling of young *P. chinense* Schneid seedlings by regulating soil nutrient cycling and the absolute abundance of key microorganisms (Gu et al., 2023). However, a critical knowledge gap persists regarding how co-application of N and P at specific ratios orchestrates a synergistic response across the plant-soil-microbe system to concurrently enhance both biomass and the biosynthesis of key medicinal compounds like berberine. Therefore, this study hypothesizes that combined N and P addition will promote the growth of *P. chinense* Schneid seedlings and the biosynthesis of berberine. Accordingly, this study analyzed physicochemical properties, enzyme activity, gene expression, microbial community composition, abundance of key microorganisms, soil metabolic compounds, biomass, and berberine content in the rhizosphere soil of *P. chinense* Schneid seedlings. The study aimed to reveal the mechanisms by which N and P supplementation promote the growth of *P. chinense* Schneid seedlings and the synthesis of berberine, providing a theoretical basis for their scientific cultivation and quality improvement, it also holds significant practical guidance for enhancing the yield and medicinal value of *P. chinense* Schneid through precision fertilization.

## 2 Materials and methods

### 2.1 Plant materials and treatment conditions

The experimental site is located on the third floor of Building A of the Arboretum at Central South University of Forestry and Technology in Changsha, Hunan Province (111°53’-114°15E’, 27°51’-28°41’N), China, with an annual average precipitation of 1361.6 mm and annual average temperature of 17.2 °C. The study area has a subtropical monsoon humid climate, with a frost-free period of 275 days and an average annual sunshine duration of 1,529.3 hours. The experimental soil is red clay, prior to the experiment, the pH value of the topsoil layer (0–20 cm) was 6.36, with SOM content of 24.28 g/kg, TN content of 1.58 g/kg, and TP content of 0.68 g/kg.

The young seedlings of *P. chinense* Schneid used in the experiment were cultivated using seeds of mature *P. chinense* Schneid native to Hunan Province. The experiment was conducted in May 2021, on an experimental plot measuring 3 m×20 m (60 m²) and seedlings were propagated using seedbeds. After a 60-day acclimatization period, 25 seedlings with similar growth rates (approximately 15 cm in height) and uniform density were selected and transplanted to the experimental field (plant spacing: 0.5 m×0.5 m) on the third floor of Building A of the Tree Science Building at Central South University of Forestry and Technology.

In this study, urea and superphosphate fertilizers were used as a source of N and P, respectively. The experiment was set up with five treatment groups, each with 5 biological replicates. The concentrations of urea and superphosphate fertilizers added were 0 g/m^2^, 10 g N/m^2^, 10 g N+5 g P/m^2^, 10 g N+10 g P/m^2^ and 10 g N+15 g P/m^2^, respectively, labeled as CK, N10, N10P5, N10P10, and N10P15, respectively. Destructive sampling was conducted on day 0 and day 90 after urea and superphosphate application. Normal field management was carried out during the experiment, with plants irrigated every two days on average and regular maintenance measures such as loosening soil and weeding.

In October 2021, destructive sampling was conducted after 90 days of fertilization, following the removal of surface litter, and the sampling depth is 20 cm. The soil around the roots was first loosened, then the *P. chinense* Schneid were uprooted, and the soil adhering to the roots was shaken off. The soil tightly bound to the root system was brushed off and collected as rhizosphere soil samples. Soil samples from the same treatment plot were uniformly mixed and sieved through a 2 mm mesh screen. All soil samples were divided into three parts: one part for determining the physical and chemical properties of soil dried at room temperature, the second part is fresh soil and placed 4°C for measuring enzyme activity, and the third part stored at -80 °C for detecting soil microorganisms, functional genes, and metabolites related to soil metabolic carbon, N, P, and sulfur. Among these, microbial analysis of soil samples, functional gene analysis, and metabolomics each employed 3, 3, and 3 technical replicates, respectively.

### 2.2 Soil physical and chemical properties determination

The soil pH value was determined using the potentiometric method, with a water-to-soil ratio of 1/2.5 (Naz et al., 2022). The SOM content was determined using the potassium dichromate method (Sun et al., 2020), TN content using the semi-micro Kjeldahl method and TP and AP content using the molybdenum antimony colorimetric method (Ma et al., 2021). The TK and AK content of the soil was determined using an inductively coupled atomic emission spectrometer (ICP-AES, Thermo Fisher, CA, USA). The NH_4_^+^-N content of the soil was determined using a Solarbio test kit (BC1510), and the NO_3_^–^-N content using a Solarbio test kit (BC0040).

### 2.3 Measurement of soil enzyme activity in the rhizosphere

Soil UR and ACP activities were measured using the sodium phenolate-sodium hypochlorite method and sodium dinitrophosphate method, respectively (Sun et al., 2020), soil BG and NAG activities were determined using the Solarbio Company test kit (BC0160 and BC4000, respectively).

### 2.4 Microbial community composition and diversity analysis

Genomic DNA was extracted from rhizosphere soil samples of *P. chinense* Schneid plants using the CTAB method, during DNA extraction, a negative control (using sterile water instead of the sample) was included to monitor for potential contamination. The purity and concentration of the soil sample DNA was detected using 1% agarose gel electrophoresis. An appropriate amount of sample was transferred to a centrifuge tube and diluted with sterile water to a concentration of 1 ng/µL. Using the primers 341F (5´-CCTAYGGGRBGCASCAG-3´) and 806R (5´-GGACTACNNGGGTATCTAAT-3´), the V3+V4 region of the 16S rRNA gene was amplified using PCR (polymerase chain reaction) technology. The PCR-amplified products were mixed into samples of equal concentration, then purified using gel electrophoresis, and finally the target bands were recovered. A library was constructed using a library preparation kit (Illumina, USA), followed by Qubit quantitative detection. After passing the quality control, sequencing was performed on the NovaSeq 6000 PE250, yielding a mean sequencing depth of 150×.

Genomic DNA was extracted from rhizosphere soil samples of *P. chinense* Schneid seedlings using the CTAB method, during DNA extraction, a negative control (using sterile water instead of the sample) was included to monitor for potential contamination. The purity and concentration of the soil sample DNA were then detected using 1% agarose gel electrophoresis. An appropriate amount of sample was transferred to a centrifuge tube and diluted with sterile water to a concentration of 1 ng/µL. PCR amplification of the ITS1-1F region of the ITS gene in soil fungi was performed using the primers ITS1-1F-F (5´-CTTGGTCATTTAGAGGAAGTAA-3´) and ITS1-1F-R (5´-GCTGCGTTCTTCATCGATGC-3´). Then PCR-amplified products were mixed into samples of the same concentration and purify them using gel electrophoresis, then cut the gel to recover the target band using the Qiagen Gel Recovery Kit from Qiagen. Subsequently, construct the library using the library construction kit (Illumina, USA), perform Qubit quantitative detection, and after passing the quality check, sequence using the NovaSeq 6000 PE250, yielding a mean sequencing depth of 150×.

### 2.5 Carbon, nitrogen, phosphorus, and sulfur functional gene chip detection

Total microbial DNA was extracted from soil samples collected from the rhizosphere of *P. chinense* Schneid roots, and the DNA concentration and purity were measured. Added the qualified microbial DNA samples and the reagents required for fluorescent quantitative PCR (quantitative real-time PCR, qRT-PCR) to a 384-well sample plate sequentially. Simultaneously, added the primers and qRT-PCR reagents to another 384-well plate as the primer plate. Subsequently, a high-throughput automated micro-dispensing device was used to add the reagents from the sample plate and primer plate into the nano-pores of the high-throughput qPCR chip. qRT-PCR reactions were performed using the SmartChip Real-Time PCR System, and fluorescent signals were detected. Amplification curves and melting curves were automatically generated. Using Canco software, we obtained detection status and Ct values (number of amplification cycles) for each gene across all samples. Data were normalized using 16S rRNA as an internal control to derive relative quantitative information for each gene in each sample. Absolute quantitative data for the 16S rRNA gene were obtained via Roche instrument analysis, and absolute quantitative data for other genes were derived through conversion. Compile Ct values for each gene across all samples into a master Ct value table using data from the SmartChip Real-Time PCR System and Canco software. Perform quality control based on the following criteria:

1. Genes with amplification efficiency below 1.8 or above 2.2 were discarded;
2. Genes showing amplification in negative controls were discarded;
3. Genes with Ct values exceeding 31 were considered non-amplified, and their Ct values in corresponding samples were discarded.

### 2.6 Metabolite analysis of rhizosphere soil

We analyzed metabolite changes in the rhizosphere soil of *P. chinense* Schneid seedlings using untargeted metabolomics (WEHEMO, Shenzhen, Guangdong, China). 100 mg of *P. chinense* Schneid rhizosphere soil sample was weighed accurately, grounded well in liquid N and transferred to an EP tube containing 500 μL of 80% methanol solution, and then shaken well using a vortex shaker. The sample was allowed to stand on an ice bath for 5 min, then centrifuged for 20 min at 15000 rpm (at 4 °C), the 300 μL supernatant was collected in a fresh EP tube, then diluted with mass spectrometry-grade water until the methanol concentration was reached to 53%, and centrifuged for 20 min at 15000 rpm (at 4 °C) and supernatant was collected in new EP tube and analyzed by UHPLC-MS/MS (Want et al., 2013).

UHPLC-MS/MS analyses were performed using a Vanquish UHPLC system (Thermo Fisher, Germany) coupled with an Orbitrap Q ExactiveTM HF mass spectrometer (Thermo Fisher, Germany). Samples were injected into a Hypersil Gold column (100×2.1 mm, 1.9 μm) using a 17-min linear gradient at a flow rate of 0.2 mL/min. The eluents for the positive polarity mode were eluent A (0.1% FA in Water) and eluent B (methanol). The eluents for the negative polarity mode were eluent A (5 mM ammonium acetate, pH 9.0) and eluent B (methanol). The solvent gradient was set as follows: 2% B, 1.5 min; 2-85% B, 3 min; 85-100% B, 10 min; 100-2% B, 10.1 min; 2% B, 12 min. Q ExactiveTM HF mass spectrometer was operated in positive/negative polarity mode with spray voltage of 3.5 kV, capillary temperature of 320 °C, sheath gas flow rate of 35 psi and aux gas flow rate of 10 L/min, S-lens RF level of 60, and aux gas heater temperature of 350 °C.

The raw data files generated by UHPLC-MS/MS were processed using the Compound Discoverer 3.1 (CD3.1, Thermo Fisher) to perform peak alignment, peak picking, and quantitation for each metabolite. The main parameters were set as follows: retention time tolerance, 0.2 minutes; actual mass tolerance, 5 ppm; signal intensity tolerance, 30%; signal/noise ratio, 3; and minimum intensity. After that, peak intensities were normalized to the total spectral intensity. The normalized data was used to predict the molecular formula based on additive ions, molecular ion peaks and fragment ions. And then peaks were matched with the mzCloud (https://www.mzcloud.org/), mzVaultand MassList database to obtain the accurate qualitative and relative quantitative results. Statistical analyses were performed using statistical software R (R version R-3.4.3), Python (Python 2.7.6 version) and CentOS (CentOS release 6.6). When data was not normally distributed, according to standardized formula: sample raw quantitation value/(The sum of sample metabolite quantitation value/The sum of QC1 sample metabolite quantitation value) was used to obtain relative peak areas; And compounds whose CVs of relative peak areas in QC samples were greater than 30% were removed, and finally the metabolites’ identification and relative quantification results were obtained (Wen et al., 2017). These metabolites were annotated using the KEGG database (https://www.genome.jp/kegg/pathway.html), HMDB database (https://hmdb.ca/metabolites) and LIPID Maps database (http://www.lipidmaps.org/).

### 2.7 Statistical analysis

The data were analyzed using SPSS software (version 22.0; IBM Corp., Armonk, NY, USA) with a significant difference level. Split-plot within a generalized linear model was employed to analyze significant differences in soil physicochemical properties, enzyme activity, and gene expression levels under combined N and P addition treatments using one-way analysis of variance (ANOVA). Non-metric multidimensional scaling (NMDS) was employed to visualize changes in bacterial and fungal community composition and clustering patterns analyzed after data normalization processing. Braden-Curtis distances between phyla and classes were assessed, and differences between NMDS groups were tested using permutation tests. RDA analysis was performed to examine the relationship between soil physicochemical properties and microbial diversity. Pearson correlation analysis and t-tests were used to integrate and visualize normalized log-transformed datasets from sequencing and metabolomics. Differential Microbial Analysis, Correlation Analysis and other plots were generated using R software (version 3.5.1; R Foundation for Statistical Computing, Vienna, Austria) and Origin software (2021 edition; OriginLab Corp., Northampton, MA, USA).

## 3 Results

### 3.1 Analysis of soil physicochemical properties and enzyme activity

Combined treatments of N10P5, N10P10 and N10P15 significantly affected pH value and contents of SOM, TN, AP and AK (**Table 1**). The pH value of the N10 group was 5.21. The pH values of N10P5, N10P10, and N10P15 group were 4.57, 4.34, and 4.58, respectively. In comparison to N10 group, combined treatments decreased pH by 12.28%, 16.70% and 12.09%, SOM contents were diminished by 31.45%, 18.81% and 29.82%, TN contents were reduced by 41.72%, 35.10% and 36.42%, TP contents were decreased by 35.90%, 28.21% and 23.08%, AP contents were lessened 40.00%, 40.00% and 40.00%, AK contents were depressed by 7.90%, 5.59% and 2.92% (**Table 1**). Compared with N10 treatment, the NH_4_^+^-N content in N10P5 experimental group was increased by 27.67%, and had no significant impact on them in N10P10 and N10P15 groups. The NO_3_^–^-N contents in N10P5 and N10P10 groups decreased by 12.72% and 14.58%, but exhibited no notable effect on this in N10P15 treatment compared with N10 experimental group. Furthermore, combined N and P addition had no obvious impact on the TK contents (**Table 1**).

**Table 1:** Effect of combined nitrogen and phosphorus addition on physicochemical properties in the rhizosphere soil of *P. chinense* Schneid seedlings.

Treatments	pH	SOM (g/kg)	TN (g/kg)	TP (g/kg)	AP (g/kg)	NH_4_^+^-N (mg/kg)	NO_3_^–^-N (mg/kg)	TK (mg/kg)	AK (mg/kg)
CK	5.29 ± 0.02a	19.48 ± 0.90a	1.17 ± 0.04b	0.33 ± 0.01b	0.02 ± 0.01c	9.31 ± 0.84b	9.55 ± 1.49b	290.20 ± 14.22a	8.08 ± 0.14ab
N10	5.21 ± 0.07a	19.62 ± 1.67a	1.51 ± 0.03a	0.39 ± 0.02a	0.05 ± 0.01a	10.41 ± 0.15ab	15.09 ± 0.47a	303.05 ± 16.20a	8.23 ± 0.12a
N10P5	4.57 ± 0.01b	13.45 ± 0.46b	0.88 ± 0.02c	0.25 ± 0.01c	0.03 ± 0.01bc	13.29 ± 1.59a	13.17 ± 1.23ab	320.55 ± 7.29a	7.58 ± 0.03c
N10P10	4.34 ± 0.03c	15.93 ± 1.19b	0.98 ± 0.04c	0.28 ± 0.01bc	0.03 ± 0.01bc	9.88 ± 0.37ab	12.89 ± 1.21ab	371.92 ± 19.66a	7.77 ± 0.12bc
N10P15	4.58 ± 0.07b	13.77 ± 0.04b	0.96 ± 0.01c	0.30 ± 0.02b	0.03 ± 0.01b	12.81 ± 1.61ab	15.42 ± 2.53a	305.84 ± 49.20a	7.99 ± 0.12ab

Data represents SD, n=3. The different letters following the numbers of the same parameter indicate significant difference (*p* < 0.05). SOM, soil organic matter; TN, total nitrogen; TP, total phosphorus; AP, available phosphorus; TK, total potassium; AK, available potassium. CK, control; N10, 10 g N/m^2^; N10P5, 10 g N+5 g P/m^2^; N10P10, 10 g N+10 g P/m^2^; N10P15, 10 g N+15 g P/m^2^.

**Table 2:** Effect of combined nitrogen and phosphorus addition on the enzyme activity in rhizosphere soil of *P. chinense* Schneid seedlings.

Treatments	UR (U/g)	ACP (U/g)	BG (U/g)	NAG (U/g)
CK	0.26 ± 0.02ab	41.33 ± 0.56a	57.11 ± 1.88a	8.00 ± 0.24b
N10	0.30 ± 0.02a	37.51 ± 1.81a	53.96 ± 3.53a	9.22 ± 0.32a
N10P5	0.21 ± 0.01bc	37.71 ± 2.74a	35.39 ± 1.93b	7.63 ± 0.14b
N10P10	0.24 ± 0.01bc	34.98 ± 2.25a	37.03 ± 2.06b	5.88 ± 0.11c
N10P15	0.21 ± 0.02c	34.94 ± 2.45a	40.69 ± 2.81b	4.93 ± 0.29d

Data represent means ± SD, n=3. The different letters following the numbers of the same parameter indicate a significant difference (*p* < 0.05). UR, urase; ACP, acid proteinase; BG, glucosidase; NAG, N-acetyl-β-D-glucosaminidase. CK, control; N10, 10 g N/m^2^; N10P5, 10 g N+5 g P/m^2^; N10P10, 10 g N+10 g P/m^2^; N10P15, 10 g N+15 g P/m^2^.

U/g means the amount of active enzyme contained in each gram of the original sample.

N10P5, N10P10 and N10P15 experimental groups, the UR activities in rhizosphere soil of *P. chinense* Schneid seedlings were reduced by 30.00%, 20.00% and 30.00%, BG activities were diminished by 34.41%, 31.38% and 24.59%, NAG activities were decreased by 17.25%, 36.23% and 46.53% compared to those in N10 group. Moreover, there was no distinct change in ACP activities between the N10 and combined N and P addition groups (**Table 2**). These results demonstrated that combined N and P additions repressed the activities of UR, BG and NAG in the rhizosphere soil of *P. chinense* Schneid seedlings, this implies that when N and P were added together, soil microbes down-regulate the production of enzymes required for decomposing complex organic polymers (e.g., cellulose and chitin), as the demand for these energy-costly processes is alleviated by the increased nutrient availability.

### 3.2 Analysis of soil microbial diversity and community composition

**Figure 1:**
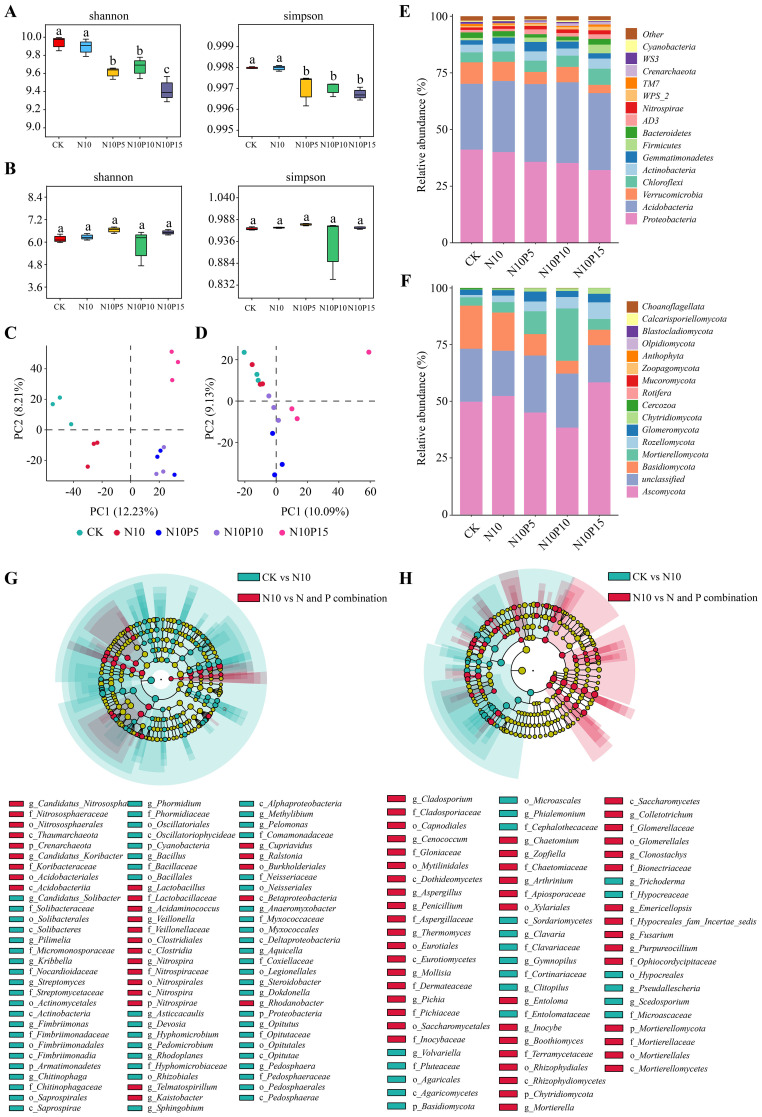
Effect of combined nitrogen and phosphorus addition on microbial diversity in rhizosphere soil of *P. chinense* Schneid seedlings. **(A)** Bacterial α-diversity; **(B)** Fungal α-diversity; **(C)** Bacterial β-diversity; **(D)** Fungal β-diversity; **(E)** Bacterial phylum; **(F)** Fungal phylum; **(G)** Speciation clade and LDA value distribution of different species of bacteria between CK and N10 with nitrogen and phosphorus addition; **(H)** Speciation clade and LDA value distribution of different species of bacteria between CK and N10 with nitrogen and phosphorus addition. CK, control; N10, 10 g N/m^2^; N10P5, 10 g N+5 g P/m^2^; N10P10, 10 g N+10 g P/m^2^; N10P15, 10 g N+15 g P/m^2^.

Combined N and P addition decreased the indices of bacterial Shannon and Simpson in rhizosphere soil, but had no obvious influence on the fungal indices compared to those in the N10 group, indicating that combined N and P addition decreased the bacterial *α*-diversity in rhizosphere soil of *P. chinense* Schneid seedlings (**Figures 1A, B**). PCoA analysis showed that the bacteria in N10P5 and N10P10 experimental groups were notably clustered, which significantly separated from N10 and N10P15 groups, but the fungi in N10P5 group were evidently separated from N10, N10P10 and N10P15 experimental groups, demonstrating that combined N and P addition influenced the microbial β-diversity in rhizosphere soil of *P. chinense* Schneid seedlings (**Figures 1C, D**).

Under combined N and P addition treatment, the *Proteobacteria* (32.1%~41.1%), *Acidobacteria* (29.1%~35.6%), *Verrucomicrobia* (3.65%~9.48%), *Chloroflexi* (4.38%~7.12%), *Actinobacteria* (3.16%~4.49%), *Gemmatimonadetes* (1.97%~4.17%), *Firmicutes* (0.48%~3.81%), *Bacteroidetes* (1.45%~2.54%), *AD3* (1.06%~2.14%) and *Nitrospirae* (1.07%~1.73%) were the top 10 bacterial phyla in term of the relative abundance, among the *Proteobacteria*, *Acidobacteria* and *Verrucomicrobia* were the dominant bacteria communities which accounted for 69.7%~79.9% of the total bacterial population in rhizosphere soil of *P. chinense* Schneid seedlings (**Figure 1E**). At the same time, compared to N10 group, the relative abundance of the *Proteobacteria* phylum decreased by 10.71%, 11.76%, and 19.72% in the N10P5, N10P10, and N10P15 treatment groups, respectively, while the relative abundance of the *Verrucomicrobia* phylum decreased by 36.61%, 19.86%, and 56.95%, respectively. Conversely, the relative abundance of the *Acidobacteria* phylum increased by 9.38%, 13.98%, and 8.35%. In N10P5, N10P10 and N10P15 experimental groups, a total of 29 differential abundance bacteria were identified, which included the *Koribacteraceae*, *Candidatus_Koribacter*, *Acidobacteriia*, *Acidobacteriales* and *Burkholderiales* belonged to *Acidobacteria* and *Proteobacteria* phyla were the top 5 bacterial taxa in terms of the relative abundance, and their relative abundance were all enhanced compared with the N10 group (**Figure 1G**).

Within fungal communities, the *Ascomycota* (38.4%~58.3%), unclassified (16.4%~25.1%), *Basidiomycota* (5.66%~19.0%), *Mortierellomycota* (3.69%~23.1%), *Rozellomycota* (1.00%~7.34%), *Glomeromycota* (2.34%~4.37%), *Chytridiomycota* (0.11%~2.50%), *Cercozoa* (0.05%~0.63%), *Rotifera* (0.00%~0.15%) and *Mucoromycota* (0.01%~0.05%) were the top 10 fungal phyla in term of the relative abundance, among the *Ascomycota*, *Basidiomycota* and *Mortierellomycota* were the primary fungal communities which accounting for 64.6%~73.8% of the total fungal population in rhizosphere soil of *P. chinense* Schneid seedlings (**Figure 1F**). At the same time, N10P5, N10P10 and N10P15 treatments reduced the relative abundance of *Basidiomycota* but enhanced the *Mortierellomycota* abundance in rhizosphere soil compared to those in the N10 group. Compared with N10 treatment, a total of 10 differential abundance fungi in N10P5, N10P10 and N10P15 groups, which the *Mortierella*, *Mortierellaceae*, *Mortierellales*, *Mortierellomycetes* and *Mortierellomycota* belonged to *Mortierellomycota* phylum were the top 5 fungal communities in terms of relative abundance, and their relative abundance was all elevated compared with the N10 group (**Figure 1H**). Collectively, these results demonstrate that combined N and P addition triggered a profound taxonomic turnover in the rhizosphere, fundamentally altering the abundance of key bacterial and fungal lineages.

### 3.3 Correlation and redundancy analysis

**Figure 2:**
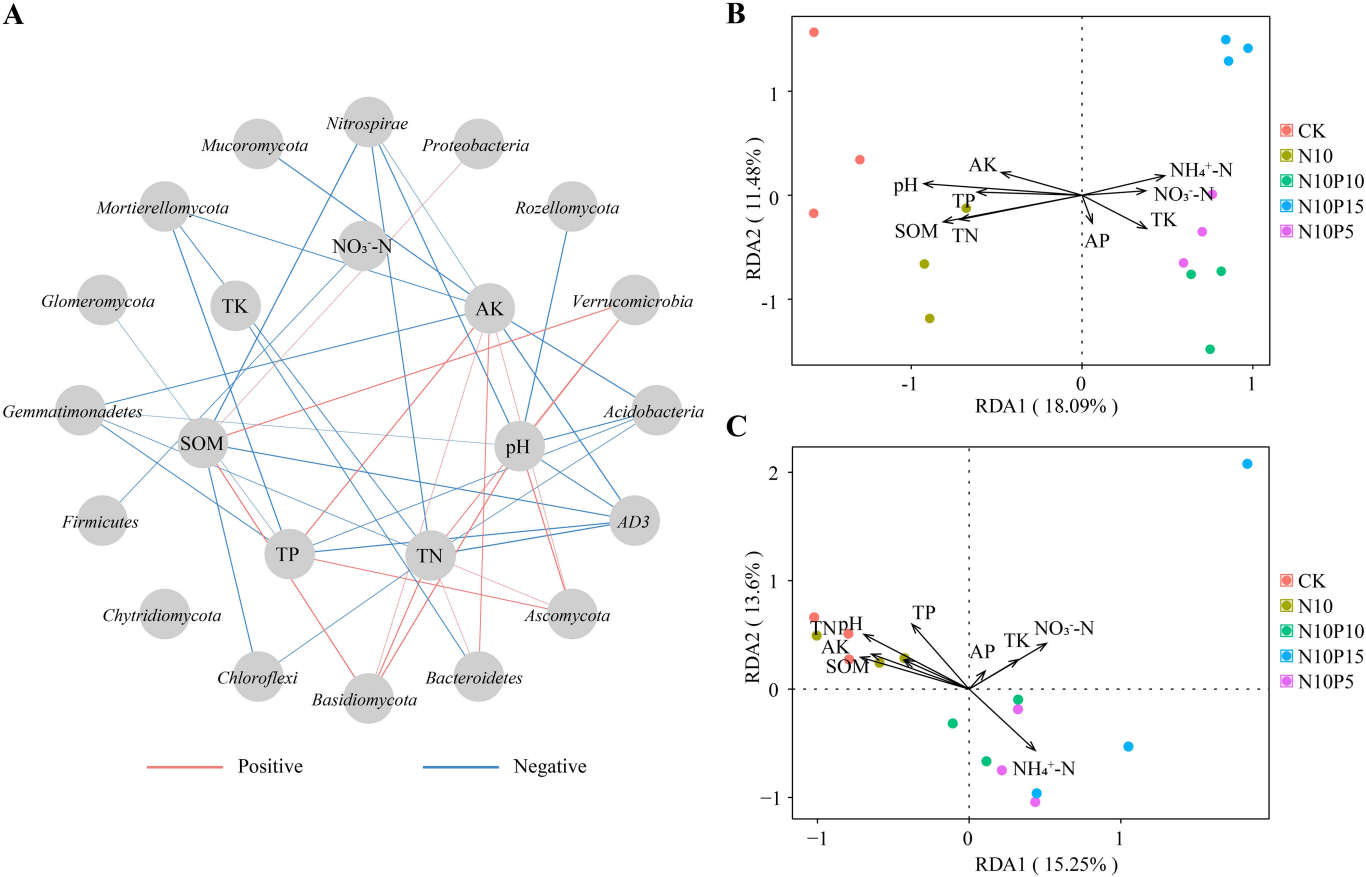
Correlation and RDA analysis between dominant microorganisms and soil physicochemical properties under nitrogen and phosphorus addition. **(A)** Correlation analysis between major bacteria and fungi with soil physical and chemical properties; **(B)** RDA analysis of major bacteria and soil physicochemical properties; **(C)** RDA analysis of major fungi and soil physicochemical properties. SOM, soil organic matter; TN, total nitrogen; TP, total phosphorus; TK, total potassium; AK, available potassium. CK, control; N10, 10 g N/m^2^; N10P5, 10 g N+5 g P/m^2^; N10P10, 10 g N+10 g P/m^2^; N10P15, 10 g N+15 g P/m^2^.

Correlation analysis showed that the *Verrucomicrobia* had a significant positive correlation with SOM (r^2^ = 0.665, *p* = 0.007), pH (r^2^ = 0.554, *p* = 0.032) and TN (r^2^ = 0.589, *p* = 0.021), the *Proteobacteria* presented a distinct positive correlation with SOM (r^2^ = 0.520, *p* = 0.047), the *Bacteroidetes* exhibited an obvious positive correlation with AK (r^2^ = 0.611, *p* = 0.016). The *AD3* displayed a notable negative correlation with SOM (r^2^ = 0.681, *p* = 0.005) and TN (r^2^ = 0.781, *p* = 0.001), the *Acidobacteria* were negatively related to pH (r^2^ = 0.652, *p* = 0.008). Moreover, the *Nitrospirae* had an apparent negative correlation with pH (r^2^ = 0.677, *p* = 0.006) and SOM (r^2^ = 0.706, *p* = 0.003), the *Chloroflexi* had an apparent negative correlation with SOM (r^2^ = 0.661, *p* = 0.007)(**Figure 2A**). Therefore, the pH (r^2^ = 0.8825, *p* = 0.001), SOM (r^2^ = 0.744, *p* = 0.002) and TN (r^2^ = 0.578, *p* = 0.007) were the main environmental factors for bacterial communities in rhizosphere soil of *P. chinense* Schneid seedlings (**Figure 2B**; **Table 3**).

**Table 3:** Results of Monte Carlo substitution test in RDA.

Environmental factor	Bacteria		Fungi	
	r^2^	*P*-value	r^2^	*P*-value
pH	0.8825	0.001	0.7527	0.001
SOM	0.7443	0.002	0.6128	0.004
TN	0.5783	0.007	0.5327	0.013
TP	0.3875	0.06	0.5201	0.015
AP	0.0892	0.563	0.0445	0.745
NH_4_^+^-N	0.2836	0.127	0.5275	0.012
NO_3_^–^-N	0.1459	0.422	0.4507	0.054
TK	0.2571	0.145	0.1887	0.282
AK	0.2738	0.145	0.2583	0.148

r^2^ means the correlation coefficient between soil physicochemical properties and OTU. pH, pH value; SOM, soil organic matter; TN, total nitrogen; TP, total phosphorus; AP, available phosphorus; AK, available potassium; TK, total potassium.

In fungal taxa, the *Cercozoa* exhibited a significant positive correlation with pH, SOM and TN, but had an obvious negative correlation with TP (*p* < 0.05). The *Basidiomycota* presented an apparent positive correlation with pH, SOM and TN, the *Ascomycota* was positively related to TP, pH and AK. The *Mortierellomycota* revealed a notable negative correlation with pH, TN, TP, SOM and AK, the *Mucoromycota* displayed a distinct negative correlation with AK. The *Rozellomycota* showed a visible negative correlation with pH (**Figure 2A**). Therefore, the pH (r^2^ = 0.753, *p* = 0.001), SOM (r^2^ = 0.613, *p* = 0.004), TN (r^2^ = 0.533, *p* = 0.013) and TP (r^2^ = 0.520, *p* = 0.015) were the dominant environmental factors for fungal taxa in rhizosphere soil of *P. chinense* Schneid seedlings (**Figure 2C**; **Table 3**). This indicates that the structure of the rhizosphere microbiome was predominantly driven by soil pH and the availability of fundamental nutrients such as carbon and nitrogen.

### 3.4 Analysis of gene expression

**Figure 3:**
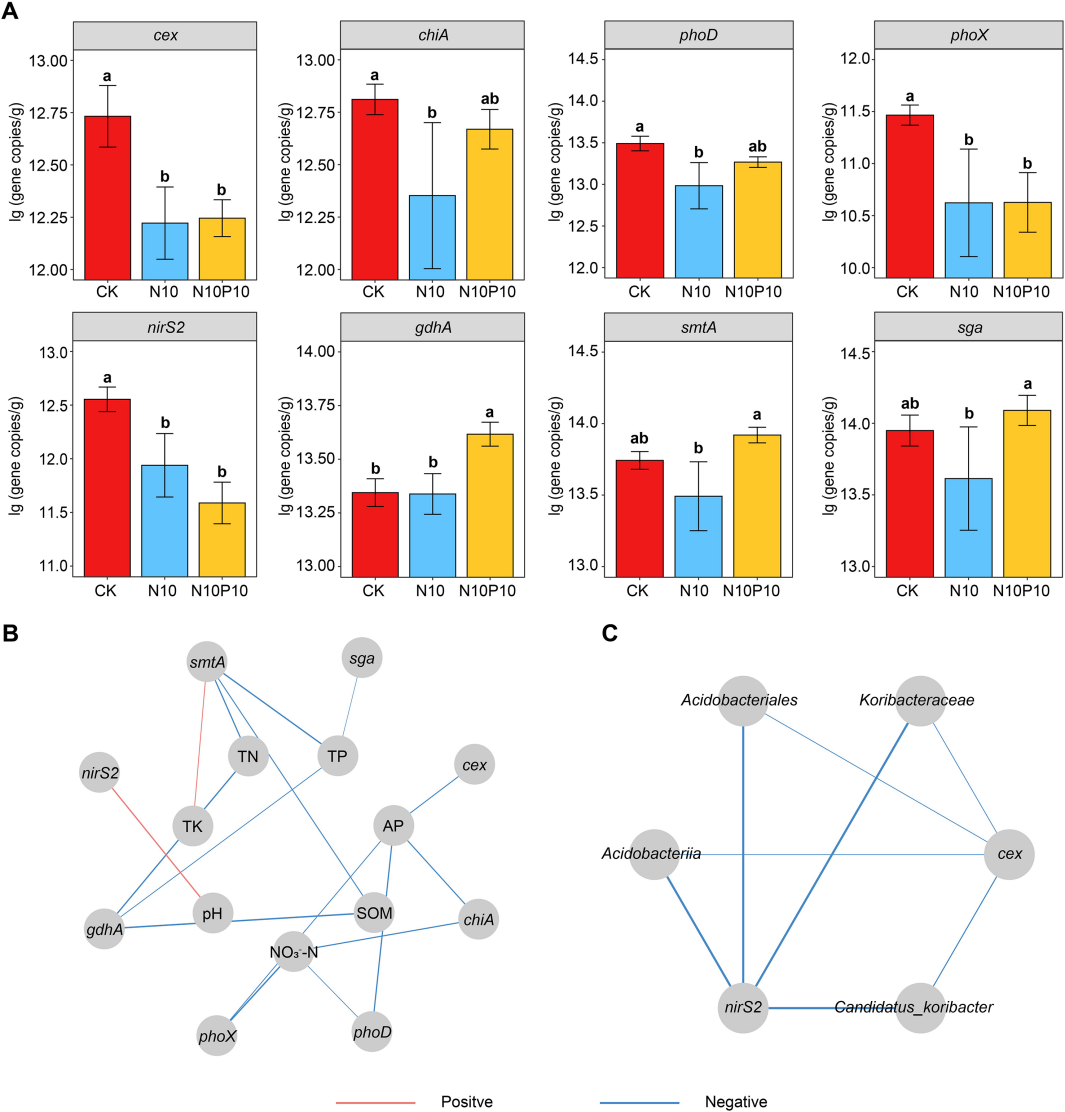
Effects of nitrogen and phosphorus addition on relative expression abundance of microbial functional genes in soil. **(A)** Microbial functional genes expression; **(B)** Correlation analysis of genes with soil physical and chemical properties; **(C)** Correlation analysis of genes with differential abundance of microorganisms. *Cex*, cellulase; *chiA*, chitinase (organic nitrogen and carbon acquisition); *phoD*, alkaline phosphatase (organic phosphorus mineralization); *phoX*, alkaline phosphatase; *nirS2*, nitrite reductase; *gdhA*, glutamate dehydrogenase (ammonium assimilation); *smtA*, metal resistance and homeostasis; *sga*, secreted β-agglutinin; SOM, soil organic matter; TN, total nitrogen; TP, total phosphorus; AP, available phosphorus; TK, total potassium; AK, available potassium. UR, urase; ACP, acid proteinase; BG, glucosidase; NAG, N-acetyl-β-D-glucosaminidase. The different letters on the bar of the same parameter indicate significant difference (*p* < 0.05). The lines marked with red and blue color represent the significant positive and negative correlations, respectively, (*p* < 0.05). CK, control; N10, 10 g N/m^2^; N10P10, 10 g N + 10 g P/m^2^.

In the N10P10 experimental group, the copies of *gdhA*, *smtA*, *sga*, *chiA* and *phoD* genes were significantly up-regulated, with fold-change values of 1.02, 1.03, 1.04, 1.03, and 1.02 increased, respectively, compared to the N10 group. However the copies of *cex*, *phoX* and *nirS2* genes had no significant change compared to the N10 group (**Figure 3A**). The correlation analysis showed that the *gdhA* and *smtA* genes presented a notable negative correlation with SOM, TN, TP, ACP, BG and NAG, and the later had an apparent positive correlation with TK. The *sga*, *chiA*, *phoX* and *phoD* genes exhibited an obvious negative correlation with TP, AP, NO_3_^–^-N and NAG, the *cex* and *nirS2* genes revealed a distinct positive correlation with ACP, BG and pH, whereas both displayed a negative correlation with *Acidobacteriales*, *Acidobacteriia*, *Koribacteraceae*, and *Candidatus_Koribacter* (**Figures 3B, C**). Overall, it indicate a fine-tuned microbial response to nutrient availability, which is orchestrated with shifts in the soil microbial community.

### 3.5 Analysis of soil metabolism

The soil metabolites in the N10 vs N10P10 experimental groups were determined and analyzed using metabolomics technology, and performed quality control and multivariate statistical analysis (**Supplementary Figure 1**). A total of 745 metabolites in N10 vs N10P10 group were identified, the explanation scores of two principal components (PC1 and PC2) were 28.4% and 21.1%, and the four biological replicates in N10P10 experimental group were significantly clustered, which notably separated from N10 treatment group, indicating that N10P10 addition had an obvious impact on the metabolites in rhizosphere soil of *P. chinense* Schneid seedlings. A total of 271 metabolites were enriched in KEGG database, which are mainly involved in the processes of metabolism (216), organismal systems (35), environmental information processing (10), drug development (4), cellular processes (4) and genetic information processing (2). In HMDB database, a total of 300 metabolites were enriched, which primarily participate in the processes of lipids and lipid-like molecules (92), organoheterocyclic compounds (54), organic acids and derivatives (39), benzenoids (35), phenylpropanoids and polyketides (27), organic oxygen compounds (22), nucleosides, nucleotides and analogues (10), alkaloids and derivatives (9) and organic N compounds (8). Within the LIPID MAPS database, total 79 metabolites were enriched, which were chiefly involved in the pathways of fatty acyls (29), sterols (17), polyketides (15), prenol lipids (12) and glycerophospholipids (5).

**Figure 4:**
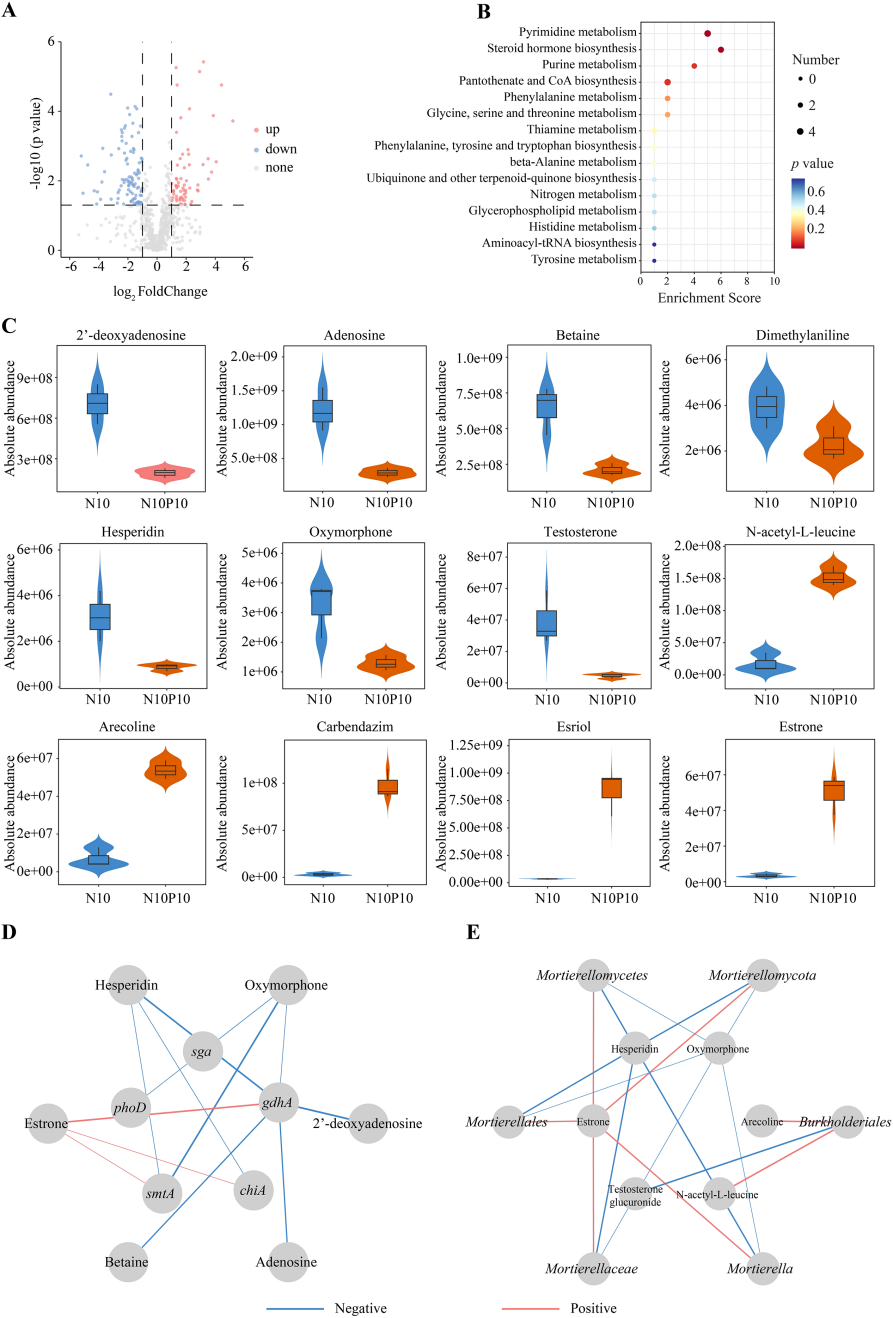
Identification and enrichment analyses and correlation analysis of DAMs in rhizosphere soil of *P*. *chinense* Schneid seedlings under nitrogen and phosphorus addition. **(A)** Volcano map of DAMs in N10 vs N10P10 group; **(B)** Enrichment analysis of DAMs in N10 vs N10P10 group; **(C)** Relative contents of DAMs in soil under nitrogen and phosphorus addition; **(D)** Correlation analysis of DAMs with gene expression; **(E)** Correlation analysis of DAMs with differentially abundant microorganisms; N10, 10 g N/m^2^; N10P10, 10 g N+10 g P/m^2^. The lines marked with red and blue colors represent the significant positive and negative correlations, respectively. (*p* < 0.05).

Based on the standard of FC>2 and *p* < 0.05, a total of 166 (64 upregulated and 102 downregulated) DAMs (differentially accumulated metabolites) in N10 vs N10P10 group were identified (**Figure 4A**), which were divided into 15 categories, and mainly enriched in the pathways of steroid hormone biosynthesis, pyrimidine metabolism, phenylalanine, tyrosine and tryptophan biosynthesis, tryptophan metabolism and phenylalanine metabolism, purine metabolism, pantothenate and CoA biosynthesis, glycine, serine and threonine metabolism, thiamine metabolism, beta-alanine metabolism, nicotinate and nicotinamide metabolism, arachidonic acid metabolism and tyrosine metabolism (**Figure 4B**). Compared with the N10 group, N10P10 treatment significantly enhanced the absolute concentrations of estriol, arecoline, carbendazim, estrone and N-acetyl-L-leucine metabolites, but decreased the absolute contents of adenosine, hesperidin, betaine, oxymorphone and 2’-deoxyadenosine metabolites (**Figure 4C**).

Correlation analysis showed that the estriol had a notable positive correlation with *smtA*, *gdhA* and *chiA* gene, in contrast, the 2’-deoxyadenosine, adenosine, and betaine exhibited an obvious negative correlation with *gdhA* gene, the oxymorphone showed a significant negative correlation with *gdhA*, *smtA*, *sga* and *phoD* genes, the hesperidin also appeared to have a distinct negative correlation with *gdhA*, *smtA* and *chiA* genes (**Figure 4D**).

The estrone displayed a significant positive correlation with *Mortierella*, *Mortierellaceae*, *Mortierellales*, *Mortierellomycetes* and *Mortierellomycota*, the arecoline and N-acetyl-L-leucine showed a significant positive correlation with *Burkholderiales*, in contrast, hesperidin and oxymorphone showed an apparent negative correlation with *Mortierella*, *Mortierellaceae*, *Mortierellales*, *Mortierellomycetes* and *Mortierellomycota*, the testosterone glucuronide indicated an evident negative correlation with *Burkholderiales* (**Figure 4E**). In summary, the rhizosphere metabolome serves as a functional nexus, directly connecting the genetic and microbial community shifts induced by combined N and P addition.

### 3.6 Regulated growth and berberine biosynthesis

Under N10P5, N10P10 and N10P15 treatments, the plant height of *P. chinense* Schneid seedlings increased by 43.0%, 83.80% and 0.95%, total fresh weight was elevated by 27.80%, 82.07% and 34.40%, root fresh weight increased by 15.40%, 36.80% and 24.0% compared to the N10 group (**Table 4**). For root nutrients, TN contents in roots increased by 22.47%, 14.61% and 8.99%, but TP contents in N10P5 and N10P15 groups decreased by 5.41% and 8.11%, and there was no change in the N10P10 group compared with the N10 treatment. Meanwhile, berberine contents in roots increased by 5.28%, 13.57% and 9.49% (**Table 4**).

**Table 4:** Effects of combined nitrogen and phosphorus additions on seedling growth and root medicinal constituents of *P. chinense* Schneid.

Treatment	Plant height	Total fresh weight	Root fresh weight	TN content in root	TP content in root	Berberine content in root
	(cm)	(g)	(g)	(g/kg)	(g/kg)	(mg/g)
CK	17.43 ± 0.86d	19.76 ± 2.29d	5.51 ± 0.89b	0.92 ± 0.09a	0.32 ± 0.01c	13.16 ± 0.86a
N10	29.45 ± 1.51c	44.06 ± 4.15c	11.97 ± 1.00a	0.89 ± 0.13a	0.37 ± 0.01a	14.96 ± 0.94a
N10P5	42.13 ± 1.52b	56.30 ± 4.09bc	13.81 ± 1.85a	1.09 ± 0.10a	0.35 ± 0.01ab	15.75 ± 1.00a
N10P10	54.13 ± 5.16a	80.22 ± 6.24a	16.37 ± 2.62a	1.02 ± 0.08a	0.37 ± 0.01a	16.99 ± 1.42a
N10P15	29.73 ± 3.62c	59.23 ± 5.63b	14.84 ± 0.75a	0.97 ± 0.03a	0.34 ± 0.01bc	16.38 ± 4.65a

Data represents means ± SD, n=3. The different letters following the numbers of the same parameter indicate significant difference (*p* < 0.05). TN, total nitrogen; TP, total phosphorus. CK, control; N10, 10 g N/m^2^; N10P5, 10 g N+5 g P/m^2^; N10P10, 10 g N+10 g P/m^2^; N10P15, 10 g N+15 g P/m^2^.

**Figure 5:**
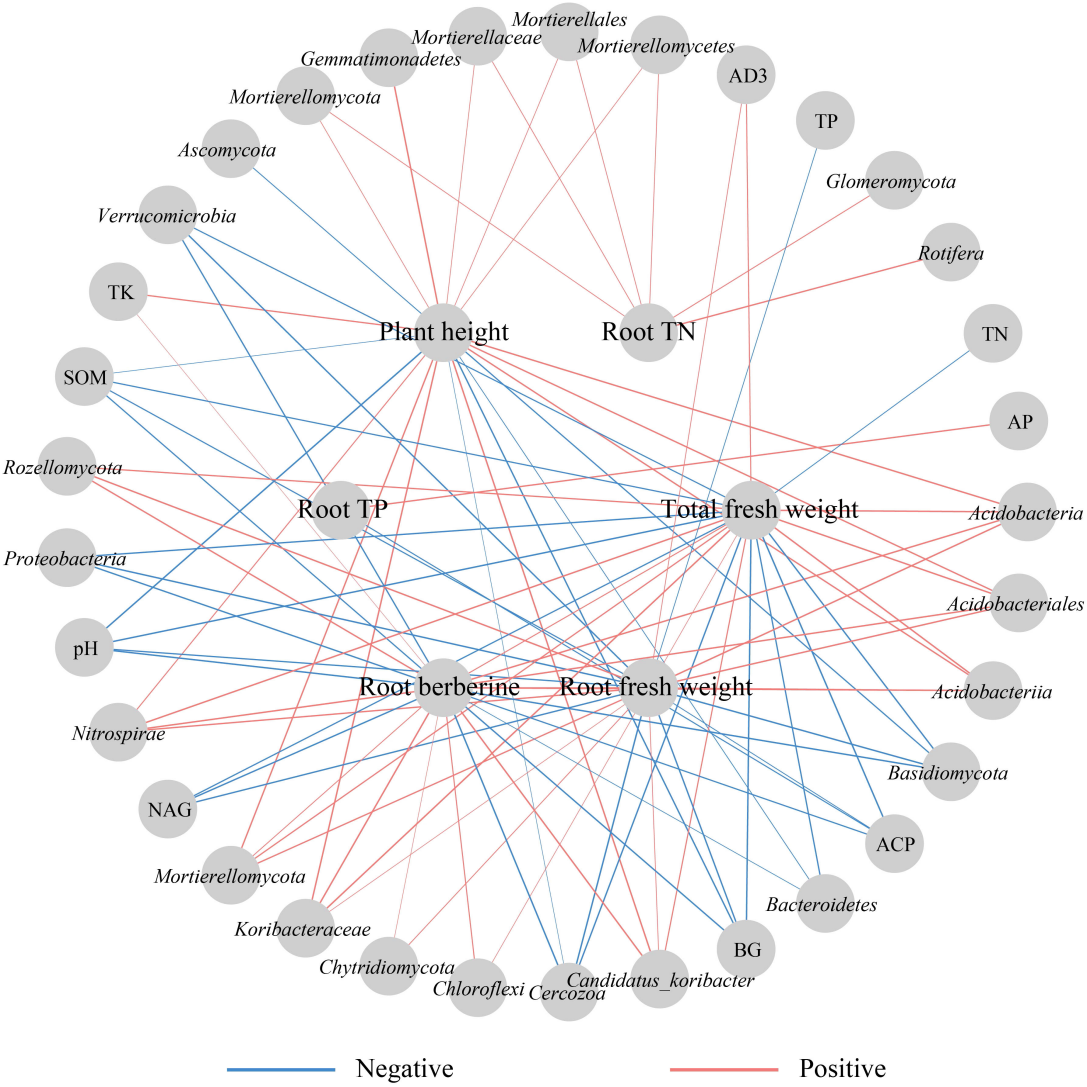
Correlation analysis between plant growth and soil physicochemical properties, enzyme activities, microbial diversity and differential abundance microorganisms. The lines marked with red and blue colors represent significant positive and negative correlations, respectively (*p* < 0.05). SOM, soil organic matter; TN, total nitrogen; TP, total phosphorus; AP, available phosphorus; TK, total potassium; AK, available potassium. UR, urase; ACP, acid proteinase; BG, glucosidase; NAG, N-acetyl-β-D-glucosaminidase.

Correlation analysis suggested that plant height was highly positively correlated with *Candidatus_Koribacter* and *Koribacteraceae*, and highly negatively correlated with pH (*p* < 0.001, **Figure 5**). Total fresh weight was strongly positively correlated with *Candidatus_Koribacter* (r=0.882), *Acidobacteria* (r=0.871), *Koribacteraceae* (r=0.939), *Acidobacteriia* (r=0.943), *Acidobacteriales* (r=0.943), and *Nitrospirae* (r=0.811), and extremely significantly negatively correlated with BG and *Basidiomycota* (*p* < 0.001, **Figure 5**). Root fresh weight was extremely highly correlated with *Candidatus_Koribacter*, *Acidobacteria*, *Koribacteraceae*, *Acidobacteriia*, *Acidobacteriales*, and *Rozellomycota*, and strongly negatively correlated with pH, *Cercozoa*, *Proteobacteria*, and *Basidiomycota* (*p* < 0.001, **Figure 5**). Root TN content was significantly positively correlated with *Rotifera*. Root TP content was significantly positively correlated with AP and negatively correlated with ACP. Root berberine content was strongly positively correlated with *Candidatus_Koribacter*, *Acidobacteria*, *Koribacteraceae*, *Acidobacteriia*, *Acidobacteriales*, and *Nitrospirae*, and highly negatively correlated with pH, *Cercozoa*, and *Basidiomycota* (*p* < 0.001, **Figure 5**). Overall, plant phenotypic and metabolic responses to nutrient addition were strongly associated with a consortium of rhizosphere microbes, highlighting the microbiome as a key driver of plant performance.

## 4 Discussion

### 4.1 Combined nitrogen and phosphorus addition reduces available nutrient content and key enzyme activity in rhizosphere soil

Our findings demonstrate that combined N and P addition induces a decline in rhizosphere nutrient availability and key enzyme activities, primarily driven by soil acidification and nutrient uptake by plants and microbes. N and P are important components of soil and significantly affect the physical and chemical properties, enzyme activity, and functional genes of plant rhizosphere soil (Nouri et al., 2014; Wang et al., 2022; Zaman et al., 2015). Previous studies have shown that N and P supplementation significantly increases nutrient content in farmland soils of the Chinese Loess Plateau. The N12P12 treatment enhanced soil enzyme activity and phospholipid fatty acid content but reduced mineralized N accumulation, indicating that N and P supplementation modulates soil physicochemical properties and enzyme activity (Kou et al., 2023). In this study, compared with N10, combined N and P treatment reduced the pH value of the rhizosphere soil of *P. chinense* Schneid, as well as the content of TN, TP, AP, AK, and SOM, while inhibiting the activity of UR, BG, and NAG, indicating that combined N and P addition reduces the levels of carbon, nitrogen, and phosphorus in the rhizosphere soil of *P. chinense* Schneid and the activity of key enzymes. The root system of *P. chinense* Schneid secretes organic acids and releases them into the soil, lowering soil pH and causing acidification, which inhibits the activity of UR, BG, and NAG in the rhizosphere soil and the rates of their carbon and N metabolic pathways, thereby reducing the content of TN, TP, AP, AK, and SOM in the rhizosphere soil (Zhang et al., 2021). On the other hand, the available nutrients in the soil are absorbed and utilized by *P. chinense* Schneid and some microorganisms, promoting their growth and proliferation, which leads to a decrease in the content of TN, TP, AP, AK, and SOM in the rhizosphere soil of *P. chinense* Schneid. This is consistent with the biomass of *P. chinense* Schneid and the abundance of some microorganisms (Wei et al., 2023; Zhang et al., 2019). In addition, combined N and P addition accelerates soil denitrification and respiration, promoting the production and release of nitrous oxide and carbon dioxide, thereby reducing the N and P contents in the rhizosphere soil of *P. chinense* Schneid (Zhang Y. et al., 2024; Zheng et al., 2023).

### 4.2 Combined nitrogen and phosphorus addition regulates microbial diversity and community structure

We found that the rhizosphere microbiome underwent a functional restructuring under combined N and P addition, where the enrichment of specific beneficial bacterial and fungal taxa was closely linked to improved plant growth. The addition of N and P regulated the proliferation of microorganisms in the rhizosphere soil, thereby reshaping microbial diversity and community structure (Hu et al., 2024; Wang et al., 2022; Zhang L. et al., 2024; Zhao et al., 2019). Nitrogen addition increased the α-diversity of rhizosphere soil bacteria in *Phragmites communis* and *Aeluropus sinensis* in the Yellow River Delta, while combined N and P addition increased the α-diversity of rhizosphere soil bacteria in *Aeluropus sinensis*, but decreased the α-diversity of rhizosphere soil bacteria in *Suaeda salsa*, while increasing the relative abundance of *Proteobacteria* and *Bacteroidetes* and decreasing the relative abundance of *Acidobacteria* (Zhang Z. et al., 2024). In this study, compared with the N10 group, the N10P5, N10P10, and N10P15 treatments reduced the α-diversity of bacteria in the rhizosphere soil of *P. chinense* Schneid, while increasing the relative abundance of the *Acidobacteria* phylum but decreasing the relative abundance of the *Proteobacteria* and *Verrucomicrobia* phyla. Additionally, bacterial communities were significantly correlated with pH, SOM, and TN, indicating that combined N and P addition reduces bacterial α-diversity and core community abundance in the rhizosphere soil of *P. chinense* Schneid by regulating soil physicochemical properties. Combined N and P addition leads to soil acidification in the rhizosphere, which favors the growth and proliferation of *Acidobacteria*, but the reduction in SOM and TN inhibits the proliferation of *Proteobacteria* and *Verrucomicrobia*, disrupting the stability and complexity of the bacterial network, thereby reducing the α-diversity of rhizosphere bacteria in *P. chinense* Schneid (Santoyo et al., 2016; Shi et al., 2023). At the same time, the reduction in pH, SOM, and TN in the rhizosphere soil is conducive to the growth of acidophilic bacteria such as *Koribacteraceae*, *Candidatus_Koribacter*, *Acidobacteriia*, *Acidobacteriales*, and *Burkholderiales*, promoting anaerobic respiration and complex organic carbon degradation in the rhizosphere soil. This, in turn, regulates the bacterial community structure and metabolic function of the rhizosphere soil of *P. chinense* Schneid promoting the growth of *P. chinense* Schneid seedlings (Hu et al., 2024; Jiang et al., 2023; Shi et al., 2023).

Compared with the N10 treatment, the N10P5, N10P10, and N10P15 treatments had no significant effect on the fungal diversity of the rhizosphere soil of *P. chinense* Schneid, while reducing the relative abundance of the *Ascomycota* and *Basidiomycota* phyla but increasing the relative abundance of *Mortierellomycota*. Additionally, the fungal community showed significant correlations with pH, SOM, TN, TP, and NH_4_^+^-N, indicating that combined N and P addition modulates the composition and relative abundance of fungal communities in the rhizosphere soil of *P. chinense* Schneid seedlings by influencing physicochemical properties. Fungi can form complex networks through hyphal formation, competing with bacteria for simple organic compounds, while also decomposing and utilizing complex organic compounds. Therefore, soil acidification had no significant effect on fungal diversity in the rhizosphere soil of *P. chinense* Schneid (Kundel et al., 2020). On the other hand, the reduction in SOM, TN, and TP content in the rhizosphere soil inhibited the proliferation of *Ascomycota* and *Basidiomycota*, hindering the degradation, conversion, and utilization of biological residues. *Mortierellomycota* has functions such as P solubilization and hormone secretion, promoting nutrient metabolism and plant growth, which is consistent with the biomass of *P. chinense* Schneid seedlings (Hu et al., 2024). What’s more, the addition of N and P increased the relative abundance of fungi *Inocybe*, *Inocybacea*e, *Aspergillaceae*, *Penicillium*, and *Aspergillus*, which we hypothesize are mainly involved in soil organic matter decomposition, biological residue degradation, and antibiotic secretion, promoting nutrient metabolism and inhibiting bacterial proliferation, thereby increasing its biomass and promoting the growth of *P. chinense* Schneid seedlings through symbiotic relationships (Chen et al., 2024; Flipphi et al., 2023; Salvatore et al., 2024). Furthermore, this is generally considered mechanistically facilitate berberine accumulation by concurrently enhancing plant nutrient acquisition, which allocates more carbon skeletons to secondary metabolism, and by enriching beneficial fungi like *Mortierellomycota* that may produce signaling molecules to upregulate the plant’s intrinsic berberine biosynthetic.

Compared to N10 group, this study demonstrated that the N10P10 treatment significantly altered the abundance of multiple microbial functional genes (*phoD*, *gdhA*, *chiA*) in the rhizosphere soil, revealing profound regulatory mechanisms underlying microbial nutrient utilization. First, the downregulation of the *phoD* gene strongly indicates that phosphorus addition effectively alleviated phosphorus limitation in soil microorganisms. As a key gene encoding alkaline phosphatase, *phoD* expression is typically negatively regulated by phosphorus availability. Thus, the elevated available phosphorus levels under N10P10 likely suppressed *phoD* expression through microbial metabolic regulatory networks, reducing energy expenditure for phosphorus acquisition (Azene et al., 2023). Second, the upregulation of the *gdhA* gene reflects enhanced microbial nitrogen assimilation. The glutamate dehydrogenase encoded by this gene is a key enzyme for microbial ammonium nitrogen assimilation (Ge et al., 2022). Its increased abundance indicates that under balanced nitrogen and phosphorus supply, the microbial community is in an active growth state, requiring more inorganic nitrogen to be converted into its own biomass. Finally, changes in the *chiA* gene reveal a shift in microbial carbon and nitrogen acquisition strategies. Chitinase not only degrades fungal cell walls (chitin) but also participates in organic nitrogen mineralization, with its expression responding to overall changes in soil carbon-nitrogen-phosphorus stoichiometry (Zhu et al., 2023). In summary, combined nitrogen and phosphorus addition does not merely alter gene abundance; rather, by modifying soil nutrient availability, it triggers precise functional reprogramming in microorganisms concerning phosphorus acquisition, nitrogen assimilation, and carbon-nitrogen cycling.

### 4.3 Combined nitrogen and phosphorus addition regulates soil metabolism

Our metabolomic analysis reveals that the rhizosphere metabolome serves as a functional nexus, through which N and P addition influences plant growth by modulating microbial abundance and the subsequent composition of bioactive metabolites, and nutrient addition regulates the relative abundance of key microorganisms in the rhizosphere soil and the vitality of plant root systems, thereby affecting soil metabolic function and metabolite abundance (Cheng et al., 2022). The addition of N and P increases the content of N containing metabolites such as amino acids, nucleosides, quaternary amines, and serotonin. Among these, serotonin shows a significant positive correlation with *Actinobacteria*, indicating that the addition of N and P promotes the accumulation of soil metabolites by regulating the abundance of key microorganisms (Baker et al., 2024). In this study, a total of 168 DAMs were identified in the N10 vs N10P10 group, among which 12 were significantly enriched in the steroid hormone biosynthesis pathway. Among these metabolites, N10P10 treatment increased the levels of estriol, arecodine, carbendazim, estrone, and N-acetyl-L-leucine, but decreased the levels of adenosine, hesperidin, betaine, oxymorphone, N, N-dimethylaniline, and 2’-deoxyadenosine. These metabolites were significantly associated with *Burkholderiales, Mortierella*, *Mortierellaceae*, *Mortierellales*, *Mortierellomycetes* and *Mortierellomycota*, indicating that combined N and P addition influences the content of major metabolites in the rhizosphere soil of *P. chinense* Schneid by regulating the abundance of key microorganisms. Under the N10P10 addition, the increased abundance of key microbial communities synergistically guided the synthesis of estriol, arecodine, carbendazim, estrone, and N-Acetyl-L-leucine in collaboration with the root system, while the decreased abundance of microbial communities inhibited the synthesis of adenosine, hesperidin, betaine, oxymorphone, N,N-dimethylaniline, and 2’-deoxyadenosine, which were then absorbed and utilized by the roots of *P. chinense* Schneid and some microorganisms, leading to a decrease in their content (Baker et al., 2024; Fan et al., 2025; Qian et al., 2020).

Among these metabolites, estriol, estrone, N-acetyl-L-leucine, and arecodine are primarily involved in processes such as steroid hormone synthesis, nitrate metabolism, and urea metabolism. These compounds are directly or indirectly absorbed and utilized by the root system, thereby enhancing leaf photosynthetic efficiency and N-P metabolic capacity, synthesizing hormones, and activating signal transduction pathways, ultimately promoting the growth of *P. chinense* Schneid seedlings (Adeel et al., 2017; Janeczko et al., 2012; Oliveira et al., 2021; Tang et al., 2024). Meanwhile, adenosine and 2’-deoxyadenosine participate in DNA synthesis, coenzyme A, and ATP synthesis metabolic processes. They are directly absorbed by the root system to enhance intracellular N and P metabolism, thereby promoting the growth of *P. chinense* Schneid seedlings (Lekberg et al., 2021). Additionally, arecodine and hesperidin exhibit antibacterial effects, and it is speculated that they play a significant role in inhibiting the proliferation of pathogenic bacteria and promoting the growth of *P. chinense* Schneid (Imperatrice et al., 2022; Ortiz et al., 2022; Pyrzynska, 2022).

### 4.4 Combined nitrogen and phosphorus addition promotes the growth of *P. chinense* Schneid

The addition of N and P regulates the distribution and metabolism of elements in plants, promoting plant growth (Jiang et al., 2019; Schleuss et al., 2020). Appropriate N and P treatment increased the content of chlorophyll a/b in Hybrid Napier Grass leaves, root length, root number, and relative growth rate (RGR), but decreased the concentration of total N in roots and phosphorus in leaves, thereby promoting growth (Pakwan et al., 2020). Compared with the N10 treatment, N and P treatments increased the total N content, berberine content, fresh root weight, total fresh weight, and plant height of *P. chinense* Schneid seedlings. Among these, the N10P10 treatment produced the highest biomass and berberine yield among treatments and was significantly correlated with the physicochemical properties of the rhizosphere soil and key microorganisms, indicating that combined N and P addition promotes the growth of *P. chinense* Schneid seedlings and the synthesis of berberine by regulating soil function. Nitrogen and P supplementation can replenish nutrient levels in the soil, with available N and P being absorbed and transported by the roots of *P. chinense* Schneid, thereby promoting the synthesis and accumulation of berberine in the roots. Consequently, it enhances photosynthetic efficiency to promote the growth of *P. chinense* Schneid seedlings, which is consistent with the levels of SOM, TN, TP, and NO_3_^–^–N in the rhizosphere soil (Pakwan et al., 2020). On the other hand, combined N and P addition reduced the activity of UR, BG, and NAG in the rhizosphere soil of *P. chinense* Schneid, while increasing the relative abundance of *Candidatus_Koribacter*, *Koribacteraceae*, *Acidobacteria*, *Acidobacteriia*, and *Acidobacteriales*. These microbial communities possess functions such as degrading organic matter in rhizosphere soil and synthesizing growth-promoting substances, thereby promoting the growth of *P. chinense* Schneid seedlings (Hu et al., 2024; Treby et al., 2020).

**Figure 6:**
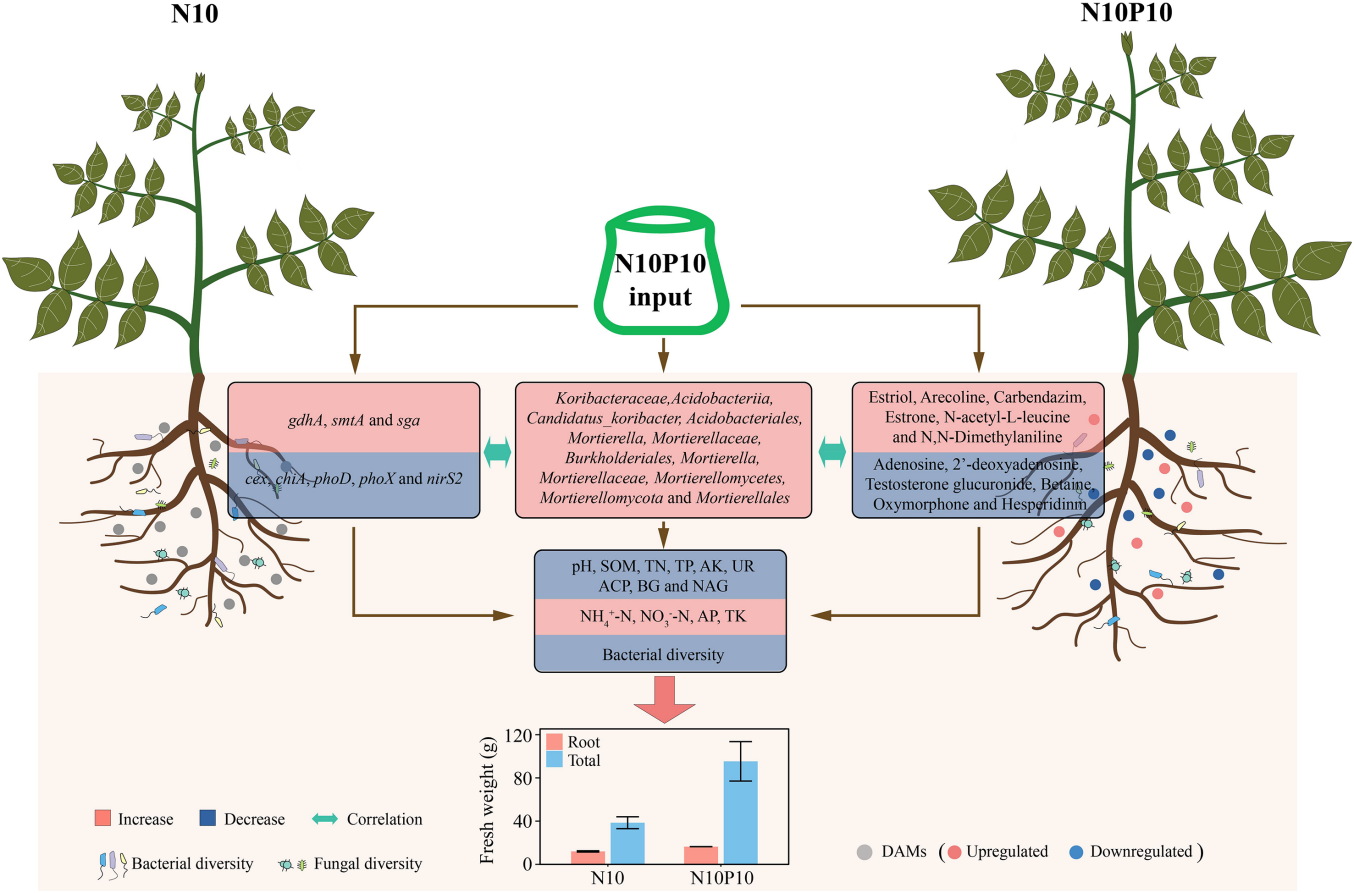
Mechanism of nitrogen and phosphorus addition to promote root growth and berberine synthesis in *P. chinense* Schneid seedlings. SOM, soil organic matter; TN, total nitrogen; TP, total phosphorus; AP, available phosphorus; TK, total potassium; AK, available potassium; UR, urase; ACP, acid proteinase; BG, glucosidase; NAG, N-acetyl-β-D-glucosaminidase; CK, control; N10, 10 gN/m^2^; N10P5, 10 gN+5g P/m^2^; N10P10, 10 gN+10 g P/m^2^; N10P15, 10 gN+15 g P/m^2^.

Based on the experimental results, we established a model to elucidate the mechanism by which N and P supplementation promotes the growth of *P. chinense* Schneid seedlings and the synthesis of berberine. Compared to the N10 group, the N10P10 treatment reduced the pH value of the rhizosphere soil of *P. chinense* Schneid seedlings, as well as the content of SOM, TN, TP, AP, AK, adenosine, hesperidin, betaine, testosterone glucuronide, oxymorphone, and 2’-deoxyadenosine content, inhibited UR, BG, and NAG activity, and reduced the expression levels of *cex*, *chi*A, *pho*D, *pho*X, and *nir*S2. It also decreased bacterial α-diversity but increased the expression levels of *gdh*A, *smt*A, and *sga*, and enhanced the abundance of the bacterial phyla *Koribacteraceae*, *Candidatus_Koribacter*, *Acidobacteriia*, *Acidobacteriales*, *Burkholderiales*, *Mortierella*, *Mortierellaceae*, *Mortierellales*, *Mortierellomycetes*, and *Mortierellomycota*, while increasing the content of estriol, arecoline, carbendazim, estrone, N-acetyl-L-leucine, N,N-dimethylaniline, and TK, thereby increasing the plant height, root fresh weight, total fresh weight, concurrently, total root nitrogen, and berberine content of *P. chinense* Schneid has also increased. Therefore, This model elucidates a core mechanistic pathway: N and P addition initiates soil acidification, which in turn selects for beneficial microbial taxa and upregulates key genes (*gdhA*, *smtA*); these shifts then drive the accumulation of growth-stimulating metabolites (e.g., estriol, N-acetyl-L-leucine), thereby directly enhancing plant biomass and berberine synthesis (**Figure 6**).

## 5 Conclusion

This study demonstrated that N10P10 group serves as a critical trigger under controlled conditions, initiating a change in the rhizosphere system. The primary event is soil acidification, which acts as an environmental filter to select for a specialized, plant-beneficial microbiome, notably enriching taxa such as *Acidobacteria* and *Mortierellomycota*. This microbial consortium functions as a living bio-fertilizer, driving a twofold mechanism: it reshapes the rhizosphere metabolome towards a growth-stimulating profile and upregulates key microbial genes involved in nitrogen assimilation. Collectively, these changes create a below-ground environment that directs the plant’s resource allocation, leading to a synergistic enhancement of both biomass (up to 82.07%) and root berberine content (by 13.57%).

Our findings provide a mechanistic framework for soil-plant-microorganism-metabolite interactions in medicinal plant systems, moving beyond correlation to reveal how fertilizer management can be leveraged to steer ecological function for agricultural benefit. The clear take-home message is that optimizing the N:P ratio, rather than simply maximizing nutrient inputs, is the decisive factor for concurrently boosting the yield and medicinal quality of *P. chinense* Schneid. For practice, this translates into a concrete fertilizer recommendation that can reduce waste and environmental impact while increasing farmer profitability. Looking forward, this mechanistic understanding opens avenues for developing microbial consortia bio-inoculants to further enhance production efficiency. Ultimately, these strategies promise a more reliable and high-quality supply of *P. chinense* Schneid for the pharmaceutical industry, strengthening the foundation of plant-derived therapeutics.

## Data Availability

The datasets presented in this study can be found in online repositories. The names of the repository/repositories and accession number(s) can be found at: NCBI-PRJNA1371413.
