# Supplementary material for: Mechanisms underlying the effects of nitrogen and phosphorus on the growth and berberine biosynthesis of Phellodendron chinense Schneid
Source: Front Plant Sci. 2025 Dec 10;16:1704035. doi: 10.3389/fpls.2025.1704035 (PMC12727914; doi:10.3389/fpls.2025.1704035)
Supplement: Supplementary file 1 [file Table1.docx]

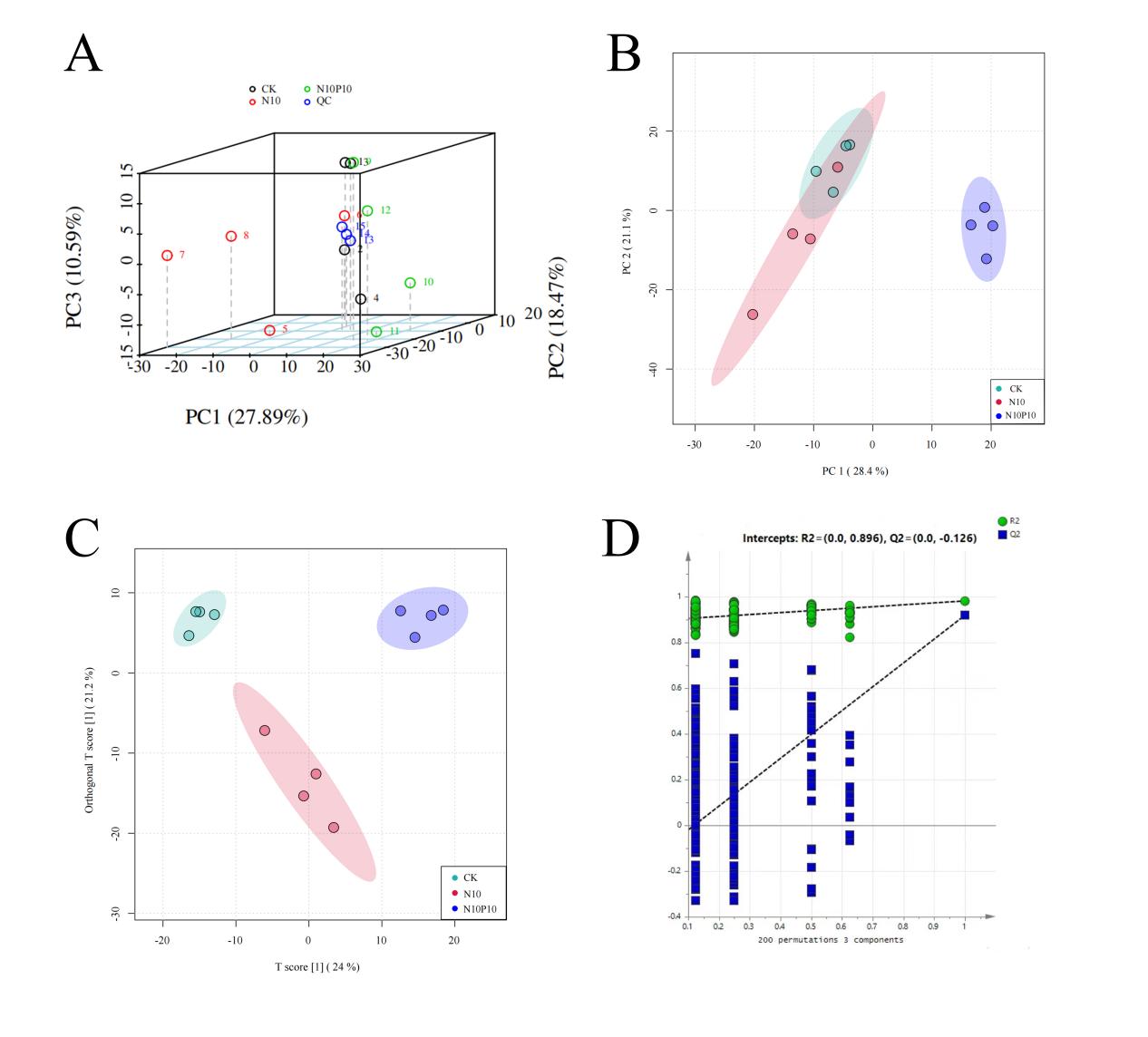


Supplementary Figure 1 Quality control of metabolomics data

(A) Quality control; (B) PCA of DAMs; (C) Analysis of DAMs based on OPLS-DA scores; (D) Cross-validation model of PLS-DA
